# Identification of partial trisomy 13q in two unrelated patients using single-nucleotide polymorphism array and literature overview

**DOI:** 10.1186/s13039-022-00608-y

**Published:** 2022-07-28

**Authors:** Jianlong Zhuang, Chunnuan Chen, Hegan Zhang, Wanyu Fu, Yanqing Li, Yuying Jiang, Shuhong Zeng, Xiaoxia Wu, Yingjun Xie, Gaoxiong Wang

**Affiliations:** 1 Prenatal Diagnosis Center, Quanzhou Women’s and Children’s Hospital, Quanzhou, People’s Republic of China; 2 Department of Neurology, The Second Affiliated Hospital of Fujian Medical University, Quanzhou, 362000 Fujian Province People’s Republic of China; 3 Department of Gynecology, Quanzhou Women’s and Children’s Hospital, Quanzhou, People’s Republic of China; 4 Department of Obstetrics and Gynecology, Guangdong Provincial Key Laboratory of Major Obstetric Diseases, The Third Affiliated Hospital of Guangzhou Medical University, Guangzhou, People’s Republic of China; 5 Key Laboratory of Reproduction and Genetics of Guangdong Higher Education Institutes, The Third Affiliated Hospital of Guangzhou Medical University, Guangzhou, People’s Republic of China; 6 Quanzhou Women’s and Children’s Hospital, Quanzhou, 362000 Fujian Province People’s Republic of China

**Keywords:** Partial trisomy 13q, Karyotype analysis, SNP array, Seizures, Developmental delay

## Abstract

**Background:**

Partial trisomy 13q is a less common chromosomal abnormality with a great clinical variability, among them, isolated partial trisomy 13q is extremely rare. Here, we report two new unrelated cases of partial trisomy 13q in Chinese families aiming to emphasize the genotype–phenotype correlation in partial trisomy 13q.

**Methods:**

Enrolled in this study were two unrelated cases of partial 13q trisomy from two families in Quanzhou region South China. Karyotpe and single-nucleotide polymorphism (SNP) array analysis were employed to identify chromosome abnormalities and copy number variants in the families.

**Results:**

A 72.9-Mb duplication in 13q14.11q34 region was identified using SNP array analysis in Patient 1 with an intellectual disability, developmental delay, seizures, gastric perforation, and other congenital malformations from a family with paternal inv(13)(p12q14.1). SNP array detection in Patient 2 revealed a 92.4-Mb duplication in 13q12.11q34 region combined with an 8.4-Mb deletion in Xq27.3q28 region with intellectual disability, developmental delay, cleft palate, and duplication of the cervix and the vagina. No chromosomal abnormality was elicited from the parents of Patient 2.

**Conclusions:**

In this study, we presented two new unrelated cases of partial trisomy 13q with variable features in Chinese population, which may enrich the spectrum of the phenotypes partial trisomy 13q and further confirm the genotype–phenotype correlation.

**Supplementary Information:**

The online version contains supplementary material available at 10.1186/s13039-022-00608-y.

## Introduction

Trisomy 13, a common chromosomal disorder characterized by severe intellectual disability and life-threatening physical abnormalities, can be detected effectively during the prenatal period using non-invasive prenatal testing [1]. Partial trisomy 13q is a rare chromosomal abnormality with variable clinical phenotypes and distinctive clinical features similar with those in trisomy 13. Partial trisomy 13q most commonly results from parental balanced translocations or inversions, and rarely from de novo [2–5]. A distinct phenotype of partial proximal and partial distal trisomy of chromosome 13q has been defined [6]. Proximal trisomy 13q usually shows strabismus, low and flat nasal bridge, persistent foetal hemoglobin (Hb), and the increased number of nuclear projects on neutrophils and other congenital malformations. On the other hand, the distal trisomy 13q often presents features of polydactyl, hemangioma, bushy eyebrows, long eyelashes, long philtrum, thin upper lip, high arched palate, ocular aberrations, and other facial malformations [7, 8].

Deletion of Xq27.3q28 region is a rare chromosomal disorder typically characterized as developmental delay, intellectual disability, and characteristic facial features [9]. Male patients show more severe clinical features than females, which resemble as Hunter syndrome and Fragile X syndrome [10, 11]. However, female patients harboring Xq27.3q28 deletion demonstrate variable clinical manifestations [9, 12, 13].

In this study, we present two new unrelated cases of partial trisomy 13q with variable features in two Chinese families. The first case involved a patient who harbored an isolated duplication in 13q14.11-qter region. The other case was a girl who harbored a large duplication of 13q12.11-qter combined with Xq27.3q28 deletion.

## Material and methods

### Subjects

Two new unrelated cases of partial trisomy 13q with variable features in two Chinese families were enrolled in this study from Fujian province, South China. Both of their parents denied consanguineous marriage and any family history of inherited disease. Both families received pretest consultation and signed the written inform, subsequently, karyotype and chromosomal microarray analysis were preformed in the patients. Ethics Committee approval was obtained from the Institutional Ethics Committee of Quanzhou Women’s and Children’s Hospital to the commencement of the study (2020No.31).

### Karyotype analysis

Approximately 2 ~  3 ml parental peripheral blood was collected from the patients and their parents for karyotype analysis. Around 20 ml amniotic fluid was obtained by amniocentesis for fetal chromosome karyotype analysis. The cultured amniotic fluid cells and peripheral blood lymphocytes were harvested using a SinochromeChromprepII automatic chromosome harvesting system according to the standard protocol (Shanghai Lechen Biotechnology Co., Ltd.) we described before [14]. Twenty metaphases were analyzed for peripheral blood karyotype and 30 metaphases were analyzed for fetal karyotype. Nomenclature of chromosomal karyotype was conducted according to ISCN 2020 [15].

### DNA extraction

Approximately 3 ~ 5 ml peripheral bloods were collected from the patients and their parents for chromosomal microarray analysis. Approximately 10 ml amniotic fluid was obtained by amniocentesis for fetal chromosomal microarray analysis. Genomics DNA were extracted from peripheral blood using QIAamp DNA Blood Kit (QIAGEN, Germany) referred to the manufacturer’s protocol (www.qiagen.com).

### SNP array detection

The single-nucleotide polymorphism array analysis was carried out using Affymetrix Cytoscan 750 K chip (Life Technologies, American) referred to the protocol described before [16]. The single-nucleotide polymorphism and copy number variants (CNVs) were analyzed using the Genotyping Console and Chromosome Analysis Suite software. The CNVs pathogenicity interpretation was conducted according to the American College of Medical Genetics (ACMG) standards and guidelines [17]. The Database of Genomic Variants (DGV) (http://dgv.tcag.ca/dgv), Online Mendelian Inheritance in Man (OMIM) (https://omim.org/), DECIPHER (https://deciphergenomics.org/) and PubMed (https: //www.ncbi.nlm.nih.gov/ pubmed/), as well as other databases, were used as reference resources.

## Results

### Patient 1

The proband in Family 1 was a girl who was the first child in this family. The patient was born full-term delivery with 50 cm (+ 0.18SD) in height and 2.45 kg ( − 2.3SD) in weight. The baby was found to have feeding difficulties and jaundice after birth. Her developmental milestone was dramatically delayed. She was unable to walk and speak until 4 years of age. A diagnosis of motor and language delay and intellectual disability was made. Gastric perforation occurred at the age of 5, for which surgical treatment was conducted. Then, seizures were observed at the age of 6, which could be controlled with antiepileptic drugs. Ultrasonography showed that the heart and kidneys were normal, but no brain MR image was available in this patient. At present, she is 8 years old, 118 cm ( − 2.0SD) tall and weighs 28 kg (+ 0.6SD), with no obvious facial dysmorphic features, except that her left ear is slightly smaller than the right one. She is now receiving compulsory education in the first grade of a primary school, which is one year behind the average children. She can read and write a little, with learning disability in arithmetic logic. In the 2nd pregnancy of Family 1, miscarriage occurred at the gestational age of 12^+^ weeks. At present, amniocentesis is carried out for prenatal diagnosis in the 3rd pregnancy of this family.

### Patient 2

The proband 2 was also a girl born as the first child of Family 2. She was delivered at the gestational age of 37 weeks, with 50 cm (+ 0.18SD) in height and 3.0 kg ( − 0.6SD) in weight. The newborn had cleft palate and duplication of the cervix and vagina. Her developmental milestone was obviously delayed. At one year of age, she was able to sit independently, but presented vertical neck instability and could not turn over at the age of 1 year. In addition, no deciduous teeth were observed. The child's psychological assessment result showed that the total development quotient was 38. At the age of 1 year and one month, she was 73.8 cm ( − 0.9SD) tall, 7.6 kg ( − 2.3SD) in weight, and her head circumference was 44.2 cm ( − 0.7SD) without obvious facial deformity. She could not walk and speak at the age of 2 years. A diagnosis of motor and language developmental delay and intellectual disability was made. No brain MRI or ultrasound images of different organs were available. At present, amniocentesis is recommended and conducted for prenatal diagnosis in the 2nd pregnancy of Family 2.

### Karyotype analysis results

**Fig. 1:**
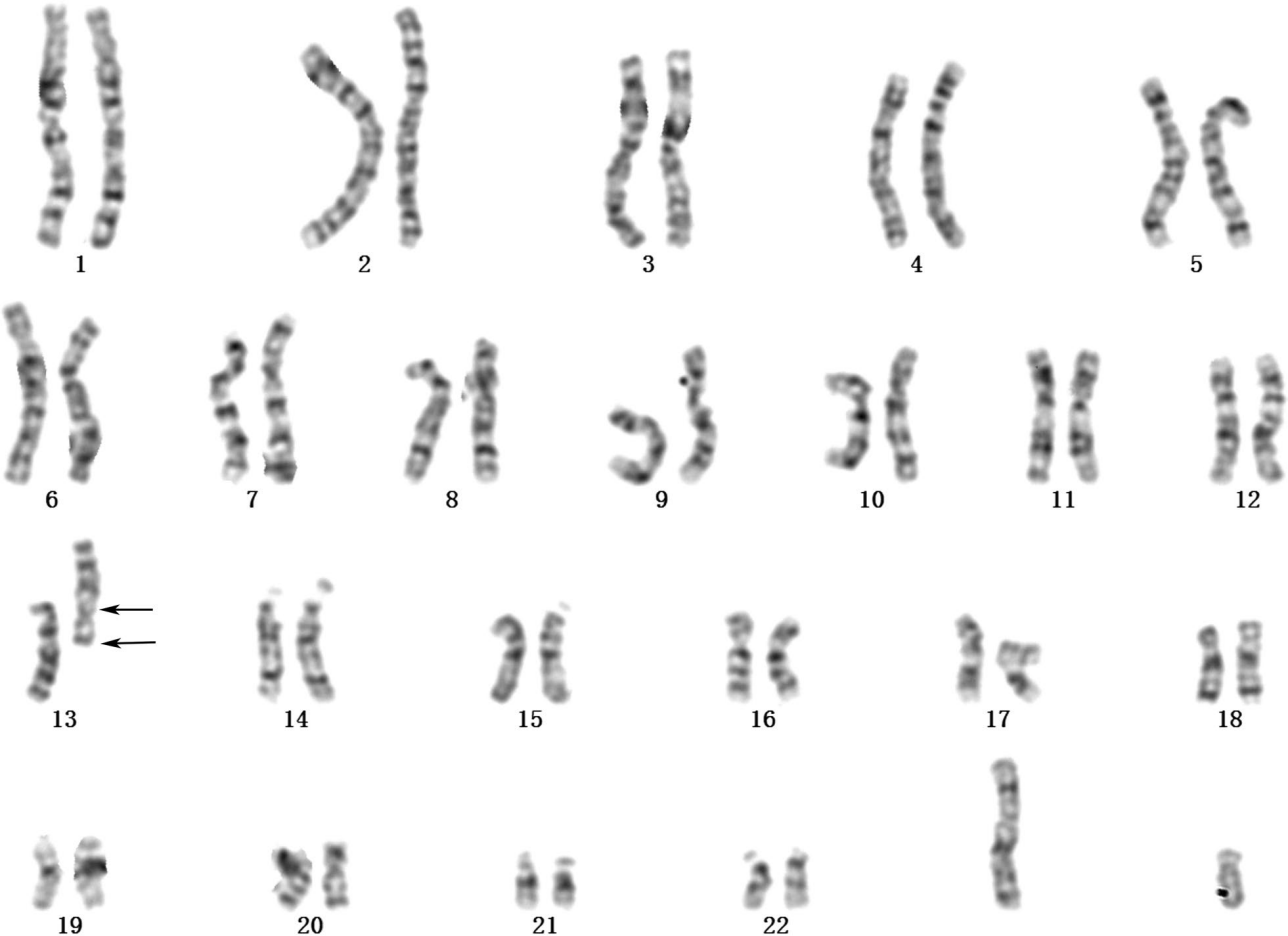
Karyotype analysis result of the proband’s father in Family 1. The arrows indicate chromosomal aberration breakpoints. The karyotype of the proband’s father was 46,XY,inv(13)(p12;q14.1)

Karyotype analysis in Patient 1 demonstrated a derivation of chromosome 13, which was described as 46,XX,rec(13)dup(13q)inv(13)(p12;q14.1)dpat (Additional file 1). Subsequent parental karyotype analysis showed that the mother’s karyotype was 46,XX and that of the father was 46,XY,inv(13)(p12;q14.1) (Fig. 1). In the 3rd pregnancy of family 1, karyotype analysis showed that the fetal karyotype was 46, X?,inv(13)(p12;q14.1).

The karyotype analysis in Patient 2 elicited an additional chromosomal material present on the long arm of chromosome X, and described as 46,XX,add(X)(q27.3) (Additional file 1). Both parents had a normal karyotype, indicating that the chromosomal abnormality in Patient 2 was a de novo structural variation. Subsequent prenatal diagnosis was carried out in the 2nd pregnancy of Family 2, showing a normal karyotype and no obvious ultrasound anomalies.

### SNP array analysis results

**Fig. 2:**
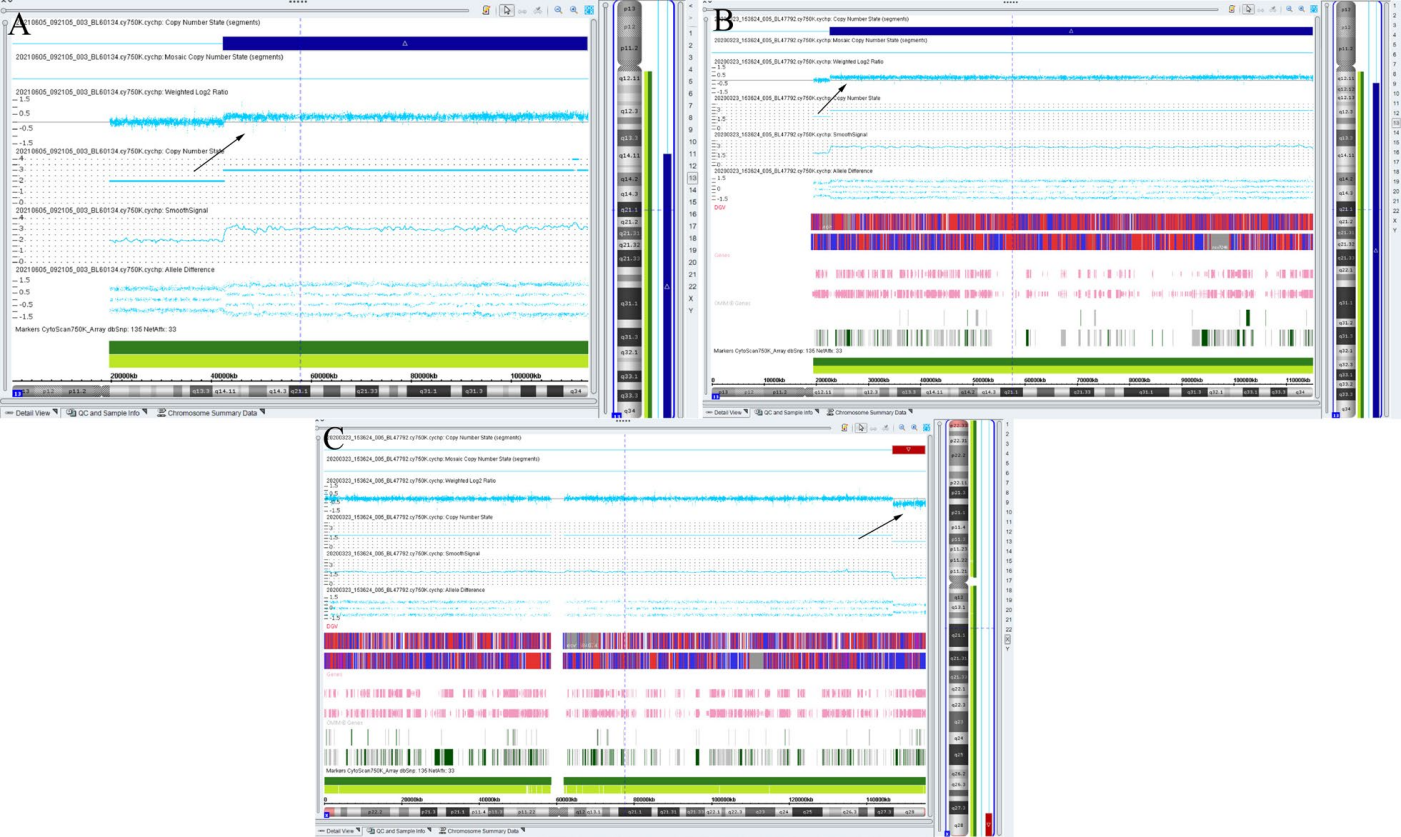
The SNP array results of Patient 1 and Patient 2. The arrows indicate chromosomal copy number variants. **A** A 72.9-Mb duplication in 13q14.11q34 region was detected in Patient 1. **B** A 92.4-Mb duplication in 13q12.11q34 region was observed in Patient 2. **C** A 8.4-Mb deletion in Xq27.3q28 region was also compounded in Patient 2

SNP array analysis results showed a large 72.9-Mb fragment duplication (arr[GRCh37]13q14.11q34(42,195,553_115,107,733) × 3) (Fig. 2A) in 13q14.11q34 region in Patient 1, which contained 178 OMIM genes. According to the ACMG guidelines, partial trisomy 13q was interpreted as pathogenic. In addition, no copy number variant was observed in the parents or in the fetus of the 3rd pregnancy of the parents.

SNP array analysis in Patient 2 showed a 92.4-Mb duplication in 13q12.11q34 region (arr[GRCh37]13q12.11q34(22,618,244_115,107,733) × 3) (Fig. 2B) associated with a 8.4-Mb deletion in Xq27.3q28 region (arr[GRCh37]Xq27.3q28(146,773,695_155,233,098) × 1) (Fig. 2C). Both variants were interpreted as pathogenic CNVs referred to ACMG guidelines. In addition, no copy number variant was observed in the fetus of the 2nd pregnancy of the parents.

## Discussion

Partial trisomy 13q is a rare chromosomal abnormality with variable clinical phenotypes*,* with few cases of isolated partial trisomy 13q are available in the literature. In this study, we report two new unrelated cases of partial trisomy 13q in two Chinese families. In addition, we reviewed the clinical findings of isolated partial trisomy 13q in partial reported cases (Table 1), and found that most variants were inherited from the parental chromosome 13 inversions, and few of them were from direct parental inheritance or from de novo.

**Table 1:** Clinical findings in partial reported cases with isolated partial trisomy 13q

References	Age/Sex	Duplication	Inheritance	Clinical features
Krygier et al. [6]	12/F	13q31.1qter	/	Learning difficulties, poor speech, facial dysmorphism, seizures, slightly developmental delay
Atack et al. [18]	12/M	13q31.1q32.3	Paternal	Developmental delay, learning disability, facial dysmorphic
Mathijssen et al. [19]	2.5/M	13q21.31q31.1	Maternal	Intellectual disability, developmental delay, dental abnormality, feeding problem, seizures, strabismus, behaviour problem
Mehra et al. [20]	7/M	13q13qter	Maternal inv(13)(p12q31)	Intellectual disability, developmental delay, learning disability, facial dysmorphic
Fraccaro et al. [21]	4/F	13q21q33.3	De novo	Trigonocephaly, low hair implantation, facial dysmorphic, bilateral clinodactyly of the fifth toes, psychomotor retardation
Habedank et al. [22]	10/M	13q22qter	Maternal inv(13)(p11q21)	Psychomotor retardation, spastic diplegia of the legs, and myoclonic and akinetic seizures, facial dysmorphic, abnormal fingers and toes
Williamson et al. [23]	34/F	13q22qter	Presumptive paternal inv(13)(p11q22)	Short stature, mentally retarded with ptosis, cleft soft palate, and polydactyly
Machado et al. [24]	Newborn/F	13q14qter	De novo	Cyanosis, hydropsy, hypotony, akinesia, and abdomen distension, short neck, and facial dysmorphism
Chen et al. [25]	Fetus	13q14.1qter	Paternal inv(13)(p12q14.1)	Intrauterine growth restriction and oligohydramnios, craniofacial dysmorphism, and camptodactyly of the right hand, bilateral subependymal cysts, left renal hydronephrosis, dilated coronary sinus with a persistent left superior vena cava, mild prominence of the left temporal horn
This study (Patient 1)	8/F	13q14.11qter	Paternal inv(13)(p12q14.1)	Intellectual disability, motor and speech developmental delay, feeding problem, gastric perforation, seizures

*F* Female; *M *Male

At present, the genotype–phenotype correlation in partial trisomy 13q has not been fully understood. Partial trisomy 13q14qter is known with variable clinical features similar to trisomy 13. Previous studies have identified isolated trisomy 13q13qter in patients with intellectual disability and facial dysmorphism inherited from their mothers who carried a pericentric inversion of chromosome 13 [20, 26]. Some studies suggested that partial trisomy 13q11q13 may not contribute to most of the features of trisomy 13; in contrast, other studies suggested the region of 13q32qter as the main region causing the clinical features of trisomy 13 [27], which was further confirmed by the study that elicited a 13q32qter duplication in a patient with several abnormalities [4]. While, a mild clinical feature was observed in an 8-year-old male harboring 13q32qter duplication [28]. In addition, another study conducted by Krygier et al. [6] demonstrated a pure 13q31.1qter duplication in a patient presenting a relatively mild phenotype, suggesting that the critical region of trisomy 13 may be placed close to somewhere in the proximal region. In addition, a patient who carried the 13q14q31 duplication exhibited the clinical features including cleft lip/palate, low set ears, depressed nasal bridge, hypertelorism, and epicanthal fold [29]. In the present study, we report two unrelated cases with 13q14q34 duplication, presenting developmental delay, intellectual disability, and other congenital malformations, which are consistent with the previous studies.

As for 13q31.3q32.3 duplication, the *GPC5* and *GPC6* are indicated as candidate genes for polydactyl [30], while some other studies containing the genes showed the absence of polydactyly [18], suggesting incomplete penetrance in these variants. Similarly, both cases in our study covered the 13q31.3q32.3 region did not have polydactyl. Several studies have shown that patients with pure partial trisomy 13q had the clinical features of cleft lip and palate [23, 29], as well as the Patient 2 in our study shared the smallest region of 13q22q31, indicating that this region may be the critical region for cleft lip and palate phenotype. The duplication of 13q31.1qter was identified in patients with seizures in previous studies [6, 22], and presented in our study as well. Therefore, it is suggested that the distal 13q region may be responsible for seizures. In addition, haemangiomas has been reported to be associated with trisomy of 13q32qter [31], no feature of haemangiomas was observed in our case at present, while, we can not rule out the occurrence of haemangiomas in the further. At present, there are few case reports describing patients who carried 13q duplication and exhibited gastrointestinal abnormalities. However, a previous study elicited internal malrotation in a patient with tetrasomy 13q31qter [32], as well as the case presented in the DECIPHER database (ID:395,925) who harbored a 15.1-Mb duplication in 13q13.2q21 region. In this study, we observed an additional feature of gastric perforation in Patient 1, but whether this is ascribed to partial trisomy 13q needs more investigation.

Patient 2 in our report also presented Xq27.3q28 deletion, which was mainly represented by intellectual disability, developmental delay, and dysmorphic facial features [9]. A previous study indicated that most of unbalanced X;autosome translocations showed a skewed inactivation of der(X) chromosome [33], moreover, a report elicited a patient with mild clinical features who had der(X)t(X;13)(p21;q32), which may ascribe to inactivation of der(X) chromosome [34]. However, another study revealed a female with a karyotype of 46,X,der(X)t(X;4)(q22;q24) and showed that a high proportion (30%) of tested autosomal genes escaped inactivation, indicating that autosomal material lacking X chromosome specific features is associated with the spreading and/or maintenance of inactivation [35]. In this study, we can not rule out the effect of trisomy 13q that contribute to the clinical features of intellectual disability and developmental delay in Patient 2. In addition, previous studies presented urogenital anomalies including hypospadias, cryptorchidism and duplicated ureter in patients with partial trisomy 13q and partial tetrasomy [28, 32], and the feature of cleft palate as well [23, 29], but no report was available in the literature to clarify the relationship between Xq27.3q28 deletion and the clinical anomalies mentioned above. Thus, we believe the urogenital anomaly of the cervix and the vagina duplication, and cleft palate observed in Patient 2 may be ascribed to partial trisomy 13q. However, more work is needed to determine the genotype–phenotype relationship as shown in Patient 2.

In conclusion, partial trisomy 13q is a rare chromosomal abnormality, especially isolated partial trisomy 13q. In this study, we presented two new unrelated cases of partial trisomy 13q with variable features, exhibiting developmental delay, intellectual disability, and other congenital malformations, which were consistent with the previous studies. In comparison with other studies, we indicated that 13q22q31 region may be critical for cleft lip and palate phenotype and the distal region of 13q may be responsible for seizures. In addition, our study enriched the phenotype spectrum of partial trisomy 13q and further confirmed the genotype–phenotype correlations.

## Supplementary Information

**Additional file 1:** Karyotype results of Patient 1 and Patient 2.

## Data Availability

The datasets used and analyzed during the current study are available from the corresponding author on reasonable request.
