# Supplementary material for: Identification of partial trisomy 13q in two unrelated patients using single-nucleotide polymorphism array and literature overview
Source: Mol Cytogenet. 2022 Jul 28;15:31. doi: 10.1186/s13039-022-00608-y (PMC9336048; doi:10.1186/s13039-022-00608-y)
Supplement: Supplementary file 1 — Additional file 1: Karyotype results of Patient 1 and Patient 2. [file 13039_2022_608_MOESM1_ESM.pdf]

A B

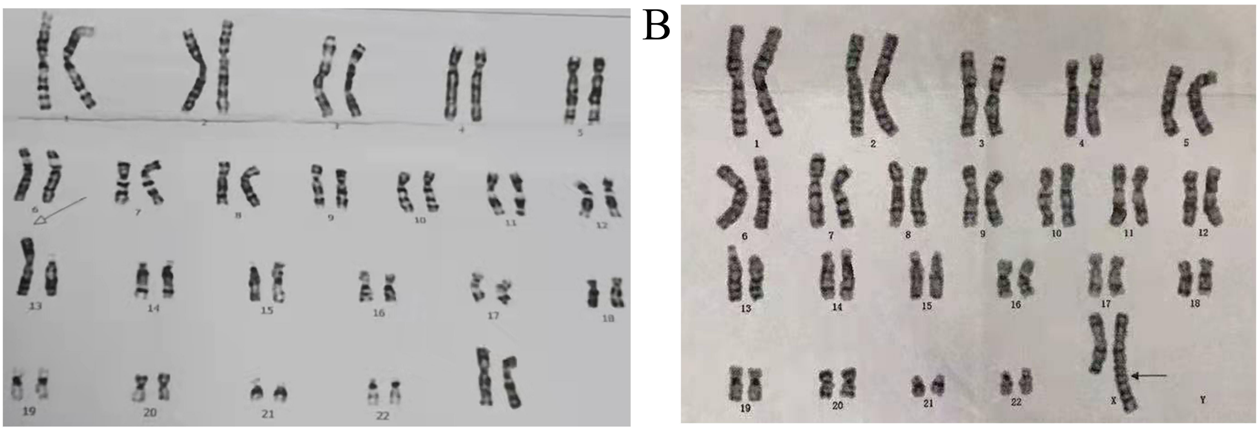

Supplemental Figure 1. The karyotype results of Patient 1 and Patient 2.

The arrows indicated the abnormal chromosomes. A: The karyotype of Patient 1 was described as 46,XX,rec(13)dup(13q)inv(13)(p12;q14.1)dpat by karyotype analysis. B: The karyotype analysis in Patient 2 elicited an additional chromosomal material present on the long arm of chromosome X, and described as 46,XX,add(X)(q27.3).
